# Mental health trajectories and family functioning among Syrian refugees in Switzerland: A five-year longitudinal study

**DOI:** 10.1016/j.jmh.2026.100431

**Published:** 2026-08-11

**Authors:** Kevin Morisod, Khoa Nguyen, Véronique S. Grazioli, Dario Spini, Elie Abboche, Chahnaz Abdulghafour, Patrick Bodenmann

**Affiliations:** a Department of Vulnerabilities and Social Medicine, Center For Primary Care and Public Health (Unisanté), Lausanne, Switzerland; b Chair of Medicine for Populations in Situation of Vulnerability, University of Lausanne (UNIL), Switzerland; c Faculty of Social and Political Sciences, University of Lausanne (UNIL), Lausanne, Switzerland; d Psychiatry and Psychotherapy Unit, Hospital Du Valais, Monthey, Switzerland

**Keywords:** Migration, Mental health, Social determinants of health, Syrian refugees, Longitudinal study

## Abstract

**Background and objectives:**

: Longitudinal evidence on mental health among refugee families across migration policy contexts remains limited. This exploratory longitudinal study describes probable psychiatric disorder prevalence, mental health-related quality of life (MHQoL), and family functioning among Syrian forced migrant families in Switzerland, comparing trajectories between families admitted through the Swiss Resettlement Program (SRP) and those entering through the standard asylum process (non-SRP).

**Methods:**

The study followed 115 participants from 34 Syrian families between 2019 and 2023. Participants completed repeated assessments of probable psychiatric disorders and MHQoL, including the SF-12 Mental Component Summary (SF-12 MCS). Family function-ing was assessed with the Family Assessment Device. Hierarchical linear models were used as secondary exploratory analyses to examine changes over time by migration pathway and family role while accounting for repeated observations within families.

**Results:**

Adults showed a higher prevalence of probable psychiatric disorders than children. Hierarchical linear models suggested that MHQoL trajectories differed by migration pathway over time, although these findings were attenuated in sensitivity analyses. Family functioning was not independently associated with MHQoL trajectories after adjustment. Mothers consistently reported lower SF-12 MCS scores than children across all adjusted models and sensitivity analyses, whereas fathers also reported lower scores, although to a lesser extent.

**Conclusion:**

Mental health patterns among Syrian forced migrant families were dynamic and heterogeneous. Differences in MHQoL trajectories by migration pathway and consistently lower MHQoL among mothers highlight the need for sustained, culturally responsive family-centered support. Larger longitudinal studies are needed to confirm these findings and clarify underlying mechanisms.

## 1 Introduction

In 2015, Europe faced a major humanitarian challenge when more than 1.25 million refugees, many fleeing the war in Syria, arrived on its shores (United Nations High Commissioner for Refugees, 2025b). Switzerland, like other European countries, adapted its asylum and healthcare systems in response. Although asylum decisions are made federally, cantons are responsible for organizing healthcare for newcomers. In the canton of Vaud, which hosts about 10% of Switzerland’s population and asylum seekers (Statistique Vaud, 2025), the reintroduction of the SRP was particularly relevant. The SRP provided immediate refugee status to vulnerable refugees resettled directly from countries of first asylum, including Lebanon and Jordan (State Secretariat for Migration, 2025).

In response, the canton of Vaud developed the Consultation for Migrant Families (CMF), a specialized family consultation service created through collaboration between the Center for Primary Care and Public Health (Unisanté) and the Children’s Hospital of Lausanne. The CMF aimed to address the complex health and psychosocial needs of resettled families and to provide tailored care for both SRP and non-SRP forced migrant families (El Ghaziri et al., 2019, El Ghaziri et al., 2021).

### 1.1 Political and legal context

Since the outbreak of the Syrian civil war in 2011, roughly one third of the Syrian population has been displaced. Switzerland reintroduced the United Nations Refugee Agency (UNHCR) resettlement program in 2015 after more than two decades of inactivity (State Secretariat for Migration, 2025). This program is designed to protect refugees who have fled their country but remain in vulnerable situations in host countries. UNHCR selects participants based on vulnerability criteria and facilitates their transfer to a safe country, where they receive refugee status and associated rights (United Nations High Commissioner for Refugees, 2025a).

The SRP selected Syrian families living in Jordan and Lebanon for resettlement, thereby bypassing the standard asylum procedure. In contrast, non-SRP families entered Switzerland through the regular asylum system, which may involve greater uncertainty regarding legal status and settlement conditions. Importantly, SRP and non-SRP groups reflect distinct migration pathways and are not assumed to be fully comparable populations; comparisons in this study are therefore descriptive rather than causal. In this study, “asylum seekers” and “refugees” distinguish between those still awaiting protection and those granted status, while “forced migrants” refers to both groups.

### 1.2 Healthcare system adaptations

The SRP created new challenges for cantonal healthcare systems, particularly in Vaud, where services had to respond to families arriving through different legal and administrative pathways. The CMF was established as a specialized service addressing medical, psychosocial, parenting, and trauma-related needs among Syrian forced migrant families (El Ghaziri et al., 2019, El Ghaziri et al., 2021, Darwiche et al., 2023). It was designed to provide culturally sensitive care and to monitor evolving family needs over time, linking federal migration policy with local healthcare delivery (El Ghaziri et al., 2021).

### 1.3 Mental health needs of Syrian forced migrants

Research on Syrian forced migrants has documented high levels of post-traumatic stress disorder (PTSD), depression, anxiety, and other mental health difficulties among adults (Hendaus et al., 2021) and children (Khamis, 2019), with similar findings reported in Europe (Schoenberger et al., 2024, Dadras and Diaz, 2024). Although living conditions and support systems differ across host contexts, psychological distress remains highly prevalent. However, less is known about how these difficulties evolve over time after resettlement within structured healthcare systems.

Mental health trajectories among forced migrants are shaped by post-migration stressors, including prolonged asylum procedures, unemployment, social isolation, and integration challenges (Laban et al., 2005). Longitudinal studies show heterogeneous patterns, with some symptoms improving and others persisting or fluctuating over time (Silove et al., 2007, Tinghög et al., 2017, Handiso et al., 2023). These findings highlight the need for longitudinal, context-sensitive descriptive research on mental health after forced displacement.

### 1.4 Factors promoting or hindering mental health

Legal status and family functioning are important contextual factors associated with forced migrants’ mental health (Ryan et al., 2009). Asylum seekers often face uncertainty and insecurity, which may increase risks of anxiety, depression, and PTSD (Laban et al., 2005). Although the present study does not focus on the asylum-seeking phase, migration pathway remains an important contextual exposure shaping post-migration experiences.

Family relationships may also influence mental health after displacement. Supportive family environments can buffer the effects of trauma, whereas family dysfunction may intensify distress (Nickerson et al., 2011). This is relevant for both adults and children, as child mental health is closely linked to parental well-being and family functioning (Khan, 1963). Family support has therefore been identified as an important resource for psychological adaptation in refugee families (Eruyar et al., 2018).

### 1.5 Study aims and exploratory questions

Despite growing evidence of mental health challenges among forced migrants, few studies have examined longitudinal patterns of probable psychiatric disorder prevalence, mental health-related quality of life, and family functioning within refugee families across migration pathways in Europe. This study prospectively followed Syrian forced migrant families living in the canton of Vaud, Switzerland. Because SRP and non-SRP families represent different migration pathways rather than comparable populations, all between-group analyses are interpreted as descriptive and exploratory. By including fathers, mothers, and children aged 9 years and older, the study also explored differences by family role and examined family functioning as a contextual factor associated with MHQoL trajectories.

Rather than estimating causal effects of the SRP, the study provides a descriptive comparison of trajectories between families admitted through the SRP and those entering through the standard asylum process. Given that SRP and non-SRP families differ in migration pathway and are not assumed to be fully comparable, all group comparisons are interpreted as exploratory.

By including fathers, mothers, and children aged 9 years and older, the study explores whether patterns differ by family role. Family functioning is examined as a contextual factor potentially associated with mental health trajectories, while acknowledging that small sample sizes, unequal group sizes, and irregular follow-up intervals limit confirmatory inference.

The study was guided by three exploratory questions. First, how do mental health-related quality of life, probable psychiatric disorder prevalence, and family functioning evolve over time among Syrian forced migrant families? Second, do descriptive trajectories differ between SRP and non-SRP families? Third, do these patterns vary by family role and family functioning? By addressing these questions, the study aims to provide longitudinal descriptive evidence to inform future research and support services for forcibly displaced families.

## 2 Methods

### 2.1 Study design

This exploratory longitudinal study examined MHQoL, probable psychiatric disorder prevalence, and family functioning among Syrian forced mi-grant families in the canton of Vaud, Switzerland. Because families admitted through the Swiss Resettlement Program (SRP) and families entering through the standard asylum process (non-SRP) differ in selection criteria, legal pathway, support conditions, and timing of observation, comparisons were interpreted descriptively rather than causally. These groups were not assumed to be comparable beyond shared service context and eligibility criteria.

The study used a family-centered design, inviting eligible fathers, mothers, and children older than eight years to participate in repeated assessments. Family functioning was examined as a potentially relevant contextual factor, rather than as a causal mechanism, given the small sample size, unequal group sizes, non-random allocation to migration pathways, and irregular follow-up intervals.

### 2.2 Participants

Eligible participants were families of Syrian origin with at least one child older than eight years who had been registered in the canton of Vaud between 2015 and 2019. The lower age limit was chosen because the study involved structured interviews and self-report questionnaires requiring sufficient cognitive, linguistic, and emotional maturity. This criterion reflected the cognitive, linguistic, and emotional demands of the assessments and excluded the direct perspectives of younger children.

Families were eligible if they were registered with local authorities and engaged with cantonal healthcare or integration services for a sufficient period to allow follow-up. Both SRP and non-SRP families meeting these criteria were invited to participate. Recruitment was facilitated through administrative data from the Etablissement vaudois d’accueil des migrants (EVAM), the Vaud institution responsible for the reception of migrants.

Families were informed about the study aims, procedures, and confidentiality protocols. Written informed consent was obtained from adult participants, and parental consent and child assent were obtained for minors. The study was approved by the local ethics committee and followed Swiss data protection regulations.

### 2.3 Data collection

Data were collected at up to five assessment waves. Baseline assessments were conducted within the first years after arrival in Switzerland, with most families arriving between 2015 and 2019 and initial interviews taking place between early 2019 and early 2020. Follow-up assessments were planned approximately annually, but timing varied according to family availability, mobility, and logistical constraints.

The second assessment generally occurred six months to one year after baseline, whereas later follow-ups were more irregular and sometimes separated by two or more years. The final available data extended into 2023 for many families. This design allowed the study to describe short- and longer-term patterns while reflecting the practical challenges of longitudinal research with forced migrant families.

Data were collected using standardized self-report questionnaires and structured interviews. Questionnaires were translated when needed by professional interpreters and administered with the support of an Arabic-speaking psychologist from the research team. Assessments took place at the Consultation for Migrant Families, participants’ homes, or community centers, depending on family preference and feasibility.

### 2.4 Measures

Mental health, family functioning, sociodemographic, and migration-related variables were collected to characterize families and describe longitudinal patterns. Detailed information on variables, scoring direction, and their role in the analyses is provided in Appendix Table 7.

#### 2.4.1 Mental health assessment

Probable psychiatric disorders were assessed using structured diagnostic interviews. Adults and adolescents aged 14 years or older completed selected modules of the Mini International Neuropsychiatric Interview (MINI), covering major depressive disorder, panic disorder, post-traumatic stress disorder, and generalized anxiety disorder (Sheehan et al., 1998). Children aged 9–13 years completed comparable MINI-Kid modules assessing major depressive disorder, panic disorder, separation anxiety, and post-traumatic stress disorder (Sheehan et al., 2010). MINI and MINI-Kid outputs were treated as categorical indicators of probable psychiatric disorder presence and used descriptively to report prevalence. Because MINI and MINI-Kid use partially overlapping but developmentally adapted modules, results across age groups are not directly comparable and were not combined into a unified diagnostic construct.

MHQoL was assessed using the Mental Component Summary (MCS) score of the 12-item Short Form Health Survey (SF-12) (Ware et al., 1996). Higher SF-12 MCS scores indicate better MHQoL. Consistent with the study protocol families (El Ghaziri et al., 2019), the SF-12 was administered only to participants aged 14 years and older who completed the self-report questionnaires. Younger children (aged 0–13 years) did not complete the SF-12; their general health was assessed using the parent-reported SF-10. Although the SF-12 provided a common measure of MHQoL across fathers, mothers, and adolescents, it has not been specifically validated in Syrian forcibly displaced populations. Findings based on the SF-12 should therefore be interpreted cautiously.

The instruments were not formally validated in this specific sample of Syrian forcibly displaced families in Switzerland. Internal consistency was therefore examined descriptively where item-level data were available. These analyses were treated as reliability checks and do not replace formal cultural validation.

#### 2.4.2 Family functioning

Family functioning was measured using the Family Assessment Device (FAD), which assesses overall family functioning, including communication, problem-solving, roles, affective involvement, and related dimensions (Byles et al., 1988). Higher FAD scores indicate more problematic perceived family functioning. The questionnaire was administered at each assessment wave to all participating parents and children aged 12 years and older. Children younger than 12 years did not complete the FAD because the instrument was not considered appropriate for this age group. Consequently, FAD data were available only for parents and older children and were unavailable for younger children.

#### 2.4.3 Sociodemographic and migration-related variables

Sociodemographic variables included age, gender, education, family composition, and length of stay in Switzerland. Migration-related variables included legal status, migration pathway, country of origin, pre-migration trauma exposure, and post-migration stressors such as housing, employment, and social support.

### 2.5 Statistical analysis

Analyses were primarily descriptive and exploratory because of the small sample size, unequal group sizes, non-random allocation to SRP and non-SRP pathways, and irregular follow-up intervals. Descriptive statistics were summarized by migration pathway, family role, and assessment wave. Means and standard deviations were reported for continuous variables, and percentages were reported for categorical variables, including probable psychiatric disorders.

Exploratory SRP versus non-SRP comparisons were conducted only when both groups contained at least three observations. Welch’s tests were used for mean comparisons, with mean differences calculated as SRP minus non-SRP. Comparisons were not reported when a test could not be estimated because of insufficient observations or zero within-group variability. Fisher’s exact tests were used for threshold-based summaries comparing the proportion of participants with SF-12 MCS scores at or below 50 and FAD scores at or above 2. These analyses were used to complement descriptive tables and figures and were not interpreted as confirmatory tests.

As a secondary exploratory analysis, hierarchical linear models were fitted using the lme4 and lmerTest packages in R (Bates et al., 2015, Kuznetsova et al., 2017). The dependent variable was SF-12 MCS. Models included random intercepts for families and for participants nested within families, accounting for repeated observations within participants and clustering within families. Because mixed-model estimates rely on large-sample assumptions, convergence warnings and singular fits were interpreted cautiously as indicators of limited information rather than model misspecification.

Assessment wave was treated as a continuous variable in the main models, with both linear and quadratic time terms included to summarize possible non-linear patterns. Model building proceeded stepwise. The null model included only random intercepts. Model 1 added migration pathway, family role, time, time squared, and SF-12 PCS. Model 2 added migration pathway-by-time interactions. Model 3 added FAD scores. Model 4 added FAD-by-time interactions. Model fit was compared using AIC, BIC, likelihood-ratio tests (Hox et al., 2017), and pseudo $R^{2}$ (Zhang, 2017). SRP families and children were used as reference categories for model parameterization only; this does not imply they represent baseline or normative groups.

Complete-case observation-level data were used for each model. Participants could contribute available observations even if they did not complete all waves, provided the variables required for the relevant model were observed. Because questionnaire eligibility differed by age, the analysis sample varied across outcomes. Specifically, the SF-12 MCS was available only for participants aged 14 years and older, whereas the FAD was administered to parents and children aged 12 years and older. Consequently, models including the SF-12 and FAD were fitted using the subset of participants eligible for those instruments and with complete data for the variables included in each model. Given the exploratory design, modest sample size, and irregular follow-up schedule, multiple imputation was not performed because the extent and pattern of missingness could not be reliably characterized.

Sensitivity analyses examined alternative model specifications, including categorical assessment wave, exclusion of observations collected more than 36 months after arrival in Vaud, and restriction to married-couple families. These analyses were interpreted descriptively and were not used for confirmatory inference.

## 3 Results

### 3.1 Sample and follow-up patterns

The study included two groups of Syrian forced migrant families living in Switzerland: families admitted through the Swiss Resettlement Program (SRP) and families who entered through the standard asylum process (non-SRP). Table 1 reports the number of families and participants observed at each of the five assessment waves, disaggregated by family role and migration pathway. Totals refer to the number of unique families or participants who contributed data at least once during the study period.

SRP families contributed more complete early follow-up data than non-SRP families. Among SRP families, participation was high during the first three waves and declined at Time 4 before partially increasing at Time 5. Follow-up was more uneven among non-SRP families, with only one family observed at Time 1 and greater variation across later waves.

**Table 1:** Number of participants surveyed across time by migration pathway and family role. Values represent available observations per wave; sample sizes vary across roles and groups.

Group	Total	Time 1	Time 2	Time 3	Time 4	Time 5
SRP						

Families	19	19	19	17	11	14
Fathers	19	19	19	17	10	13
Mothers	19	19	19	17	10	14
Children	43	33	38	33	13	18

Non-SRP						

Families	15	1	8	10	5	8
Fathers	12	1	5	6	4	6
Mothers	15	1	8	10	5	8
Children	18	2	11	10	3	6

Because of these unequal observation patterns, particularly the limited number of non-SRP observations at Time 1, between-group comparisons should be interpreted descriptively. Missing observations were handled as described in Section 2.5, and sensitivity analyses were used to assess robustness to differences in follow-up timing and sample composition.

### 3.2 Reliability of measures

Descriptive internal consistency estimates are presented in Table 2. The FAD showed good internal consistency in the present sample, with Cronbach’s α = 0.843 and McDonald’s ω = 0.868. Internal consistency was calculated using all available FAD item responses pooled across assessment waves because the same 12-item General Functioning subscale, response options, and scoring procedures were used consistently throughout the study.

The MINI and MINI-KID are structured diagnostic interviews designed to generate DSM-aligned diagnostic classifications using algorithm-based decision rules with skip patterns rather than psychometric scale scores. Consequently, internal consistency indices such as Cronbach’s α or KR-20 are not conceptually appropriate are not appropriate and are not reported. MINI and MINI-KID outputs were treated as categorical indicators of probable psychiatric dis-order presence and used descriptively to report prevalence across migration pathway and family role.

**Table 2:** Descriptive internal consistency estimates.

Measure/module	Items	Observations	Cronbach’s α/KR-20	McDonald’s ω
FAD	12	342	0.843	0.868

*Note:* FAD = Family Assessment Device. Cronbach’s α and McDonald’s ω are reported for the FAD using available item-level data pooled across all assessment waves.

Likewise, internal consistency was not evaluated for the SF-12. The primary outcome was the MCS, a weighted summary score derived from the SF-12 scoring algorithm rather than a unidimensional psychometric scale. Accordingly, conventional internal consistency coefficients such as Cronbach’s α are not considered appropriate for the MCS.

### 3.3 Psychiatric disorder prevalence

Table 3 presents the prevalence of probable psychiatric disorders among Syrian forced migrant families in Switzerland, stratified by migration pathway and family role. Disorders assessed included major depressive disorder (MDD), panic disorder (PD), post-traumatic stress disorder (PTSD), and generalized or separation anxiety (GA/SA), with both past and present episodes reported. Because subgroup sizes were small, particularly in some non-SRP strata, percentages should be interpreted descriptively and with caution.

Overall, probable psychiatric disorders were common among adults in both SRP and non-SRP families. High proportions of fathers and mothers met criteria for probable MDD, panic disorder, PTSD, or generalized/separation anxiety. Children showed lower observed prevalence across most disorders, although these estimates were based on small subgroup sizes and should not be interpreted as evidence of absence of need.

**Table 3:** Prevalence of probable psychiatric disorders by family role and migrationtype.

Disorder	SRP			Non-SRP		
	Fathers	Mothers	Children	Fathers	Mothers	Children
	(N = 19)	(N = 19)	(N = 43)	(N = 12)	(N = 15)	(N = 18)
Past MDD	63.16	68.42	9.09	75.00	73.33	15.79
Present MDD	47.37	63.16	4.55	75.00	53.33	5.26
Past PD	42.11	47.37	2.27	50.00	40.00	10.53
Present PD	31.58	47.37	4.55	41.67	33.33	0.00
Past PTSD	57.89	52.63	0.00	58.33	60.00	10.53
Present PTSD	52.63	42.11	0.00	58.33	46.67	10.53
Past GA/SA	52.63	57.89	0.00	50.00	53.33	0.00
Present GA/SA	47.37	57.89	0.00	58.33	53.33	15.79

*Note:* Values are percentages per subgroup. MDD = Major Depressive Disorder; PD = Panic Disorder; PTSD = Post-Traumatic Stress Disorder; GA/SA = Generalized Anxiety/Separation Anxiety. Percentages are based on small subgroup sizes and should be interpreted descriptively.

**Fig. 1:**
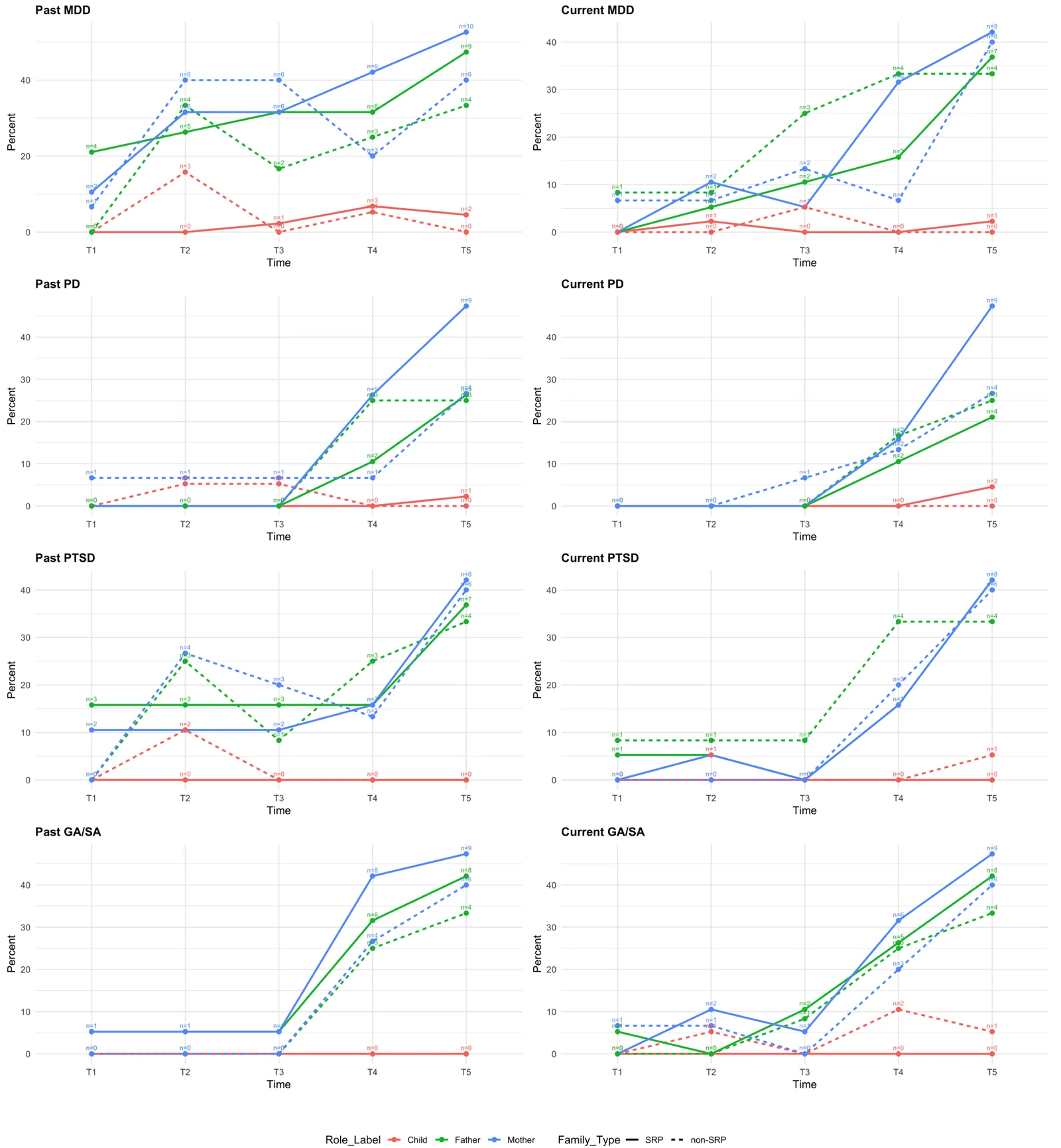
Descriptive evolution of the prevalence (%) of probable psychiatric disorders across five assessment waves, stratified by family role and migration pathway (SRP vs. non-SRP). Numbers displayed at each point indicate the number of participants with a non-missing observation and meeting criteria for the respective disorder at each wave.

Fig. 1 illustrates the descriptive evolution of probable psychiatric disorders across the five assessment waves by migration pathway and family role. Visual inspection suggests higher prevalence among adults than children throughout follow-up. In some later waves, mothers, particularly in SRP families, appeared to show high prevalence across several disorders. However, these patterns are descriptive and should not be interpreted as statistically confirmed differences between roles, groups, or waves given very small cell sizes.

Taken together, Table 3 and Fig. 1 suggest a substantial burden of probable psychiatric disorders among adult Syrian forced migrants, alongside lower observed prevalence among children. These descriptive findings provide context for the subsequent analyses of MHQoL using SF-12 MCS scores.

### 3.4 Mental health-related quality of life (MHQoL)

Table 4 presents mean SF-12 MCS scores, with standard deviations, for fathers, mothers, and children in SRP and non-SRP families across the five assessment waves. As summarized in Appendix Table 7, the SF-12 MCS was used as the measure of MHQoL and was the main dependent variable in the exploratory longitudinal models. Higher SF-12 MCS scores indicate better MHQoL. Because subgroup sizes varied across waves and were particularly small for some non-SRP role-by-time groups, these values should be interpreted descriptively.

To complement the descriptive means, exploratory SRP versus non-SRP comparisons were conducted within each assessment wave and family role when both groups contained sufficient observations. Welch’s tests compared mean SF-12 MCS scores, and Fisher’s ex-act tests compared the proportion of participants with SF-12 MCS scores at or below the reference value of 50. Comparisons that could not be estimated because of insufficient observations or zero within-group variability are reported descriptively without inferential statistics. Detailed results are reported in Table 8, Table 9.

**Table 4:** Means (with standard deviation) of SF-12 mental component summary scores across time by family role and migration type.

T	SRP			Non-SRP		
	Fathers	Mothers	Children	Fathers	Mothers	Children
1	56.37(6.50)	53.17(7.63)	57.49(5.17)	22.12(NA)	31.03(NA)	–
2	52.24(9.48)	47.29(12.39)	51.89(6.34)	40.11(12.66)	47.23(11.37)	45.41(11.04)
3	51.24(12.73)	42.43(13.31)	54.27(7.62)	45.44(20.10)	42.59(14.07)	56.66(1.18)
4	43.70(10.56)	40.95(9.65)	57.92(5.68)	54.14(17.32)	45.35(8.67)	42.72(NA)
5	39.03(8.82)	41.76(7.00)	51.52(11.22)	33.89(10.32)	41.06(3.33)	52.28(7.43)

*Note:* Values are means (standard deviation). Higher SF-12 MCS scores indicate better MHQoL. NA indicates that a standard deviation could not be calculated because only one observation was available; – indicates that no children were surveyed in the non-SRP group at Time 1.

SRP and non-SRP participants showed different descriptive patterns in SF-12 MCS scores at some waves, but these patterns were inconsistent across family roles and assessment waves. The largest exploratory mean difference was observed among fathers at Time 2, where SRP fathers had higher mean SF-12 MCS scores than non-SRP fathers by 12.13 points. However, the confidence interval was wide and included zero, and the Welch test did not reach conventional statistical significance (p=0.099). Among mothers, mean differences were small at most waves.

Threshold comparisons showed similar heterogeneity. At Time 2, a higher proportion of non-SRP fathers and children had SF-12 MCS scores at or below 50 than SRP fathers and children, but Fisher’s exact tests did not indicate statistically robust group differences. At Time 5, high proportions of parents in both groups had scores at or below 50, suggesting substantial adult MHQoL difficulties in later follow-up without supporting a simple or consistent SRP versus non-SRP difference.

**Fig. 2:**
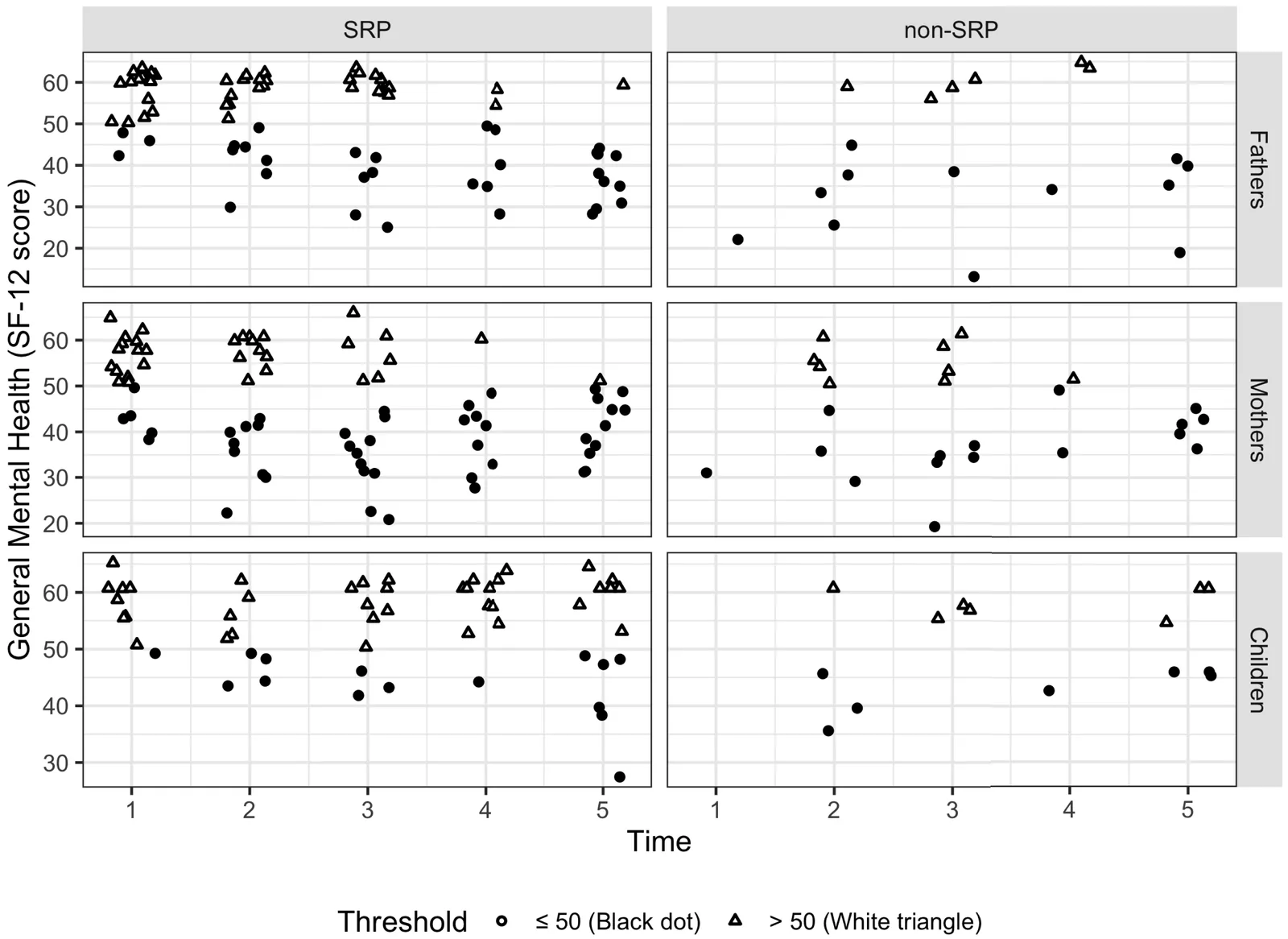
Individual SF-12 MCS scores across time. Higher scores indicate better MHQoL; scores at or below 50 indicate poorer-than-average MHQoL relative to the reference value.

Fig. 2 displays individual SF-12 MCS scores over time, grouped by migration pathway and family role. The scatterplot illustrates the variability underlying the descriptive means and exploratory comparisons. Visual patterns reflect raw individual-level variability over time and should not be interpreted as evidence of group differences, trajectories, or temporal effects.

Overall, the descriptive means, exploratory mean comparisons, and threshold comparisons suggest heterogeneous MHQoL patterns by migration pathway, family role, and time. This heterogeneity is consistent with the instability observed in model-based estimates of SRP/non-SRP effects in the hierarchical linear models. The SF-12 MCS results should be interpreted separately from MINI-based psychiatric disorder prevalence, as the former measures continuous health-related quality of life while the latter classifies probable DSM-aligned diagnostic categories.

### 3.5 Family functioning

Table 5 presents mean Family Assessment Device (FAD) scores, with standard deviations, for fathers, mothers, and children in SRP and non-SRP families across the five assessment waves. As summarized in Appendix Table 7, higher FAD scores indicate more problematic perceived family functioning. Because subgroup sizes varied across waves and were particularly small for some non-SRP role-by-time groups, these values should be interpreted descriptively.

To complement the descriptive means, exploratory SRP versus non-SRP comparisons were conducted within each assessment wave and family role when both groups contained sufficient observations. Welch’s tests compared mean FAD scores, and Fisher’s exact tests compared the proportion of participants with FAD scores at or above the reference value of 2. Comparisons that could not be estimated because of insufficient observations or zero within-group variability are reported descriptively without inferential statistics. Detailed results are reported in Table 10, Table 11.

**Table 5:** Means (with standard deviation) of FAD scores across time by family role and migration type.

T	SRP			Non-SRP		
	Fathers	Mothers	Children	Fathers	Mothers	Children
1	2.39(0.16)	2.43(0.13)	2.45(0.12)	2.25(NA)	2.50(NA)	2.25(0.00)
2	2.42(0.10)	2.42(0.16)	2.46(0.16)	2.37(0.17)	2.49(0.21)	2.48(0.25)
3	2.44(0.14)	2.39(0.17)	2.45(0.14)	2.56(0.42)	2.55(0.39)	2.55(0.12)
4	1.69(0.15)	1.56(0.33)	1.75(0.27)	2.00(0.38)	1.89(0.05)	1.92(NA)
5	1.46(0.18)	1.46(0.28)	1.59(0.52)	1.44(0.14)	1.46(0.14)	1.71(0.51)

*Note:* Values are means (standard deviation). Scores range from 1 to 4, with higher scores indicating more problematic family functioning. NA indicates that a standard deviation could not be calculated because only one observation was available.

During the first three waves, mean FAD scores were generally above the reference value of 2 in both SRP and non-SRP families, suggesting more problematic perceived family functioning in earlier assessments. At Times 4 and 5, mean FAD scores were lower in most family-role groups and generally below 2, suggesting less problematic perceived family functioning later in follow-up.

Exploratory mean comparisons indicated that SRP and non-SRP differences varied by role and wave. Most comparisons did not indicate statistically robust group differences. Some comparisons suggested lower FAD scores among SRP participants, such as mothers at Time 4, but this comparison involved only three non-SRP mothers and should therefore be interpreted cautiously. Overall, these comparisons do not support a consistent SRP versus non-SRP difference in family functioning across roles and waves.

**Fig. 3:**
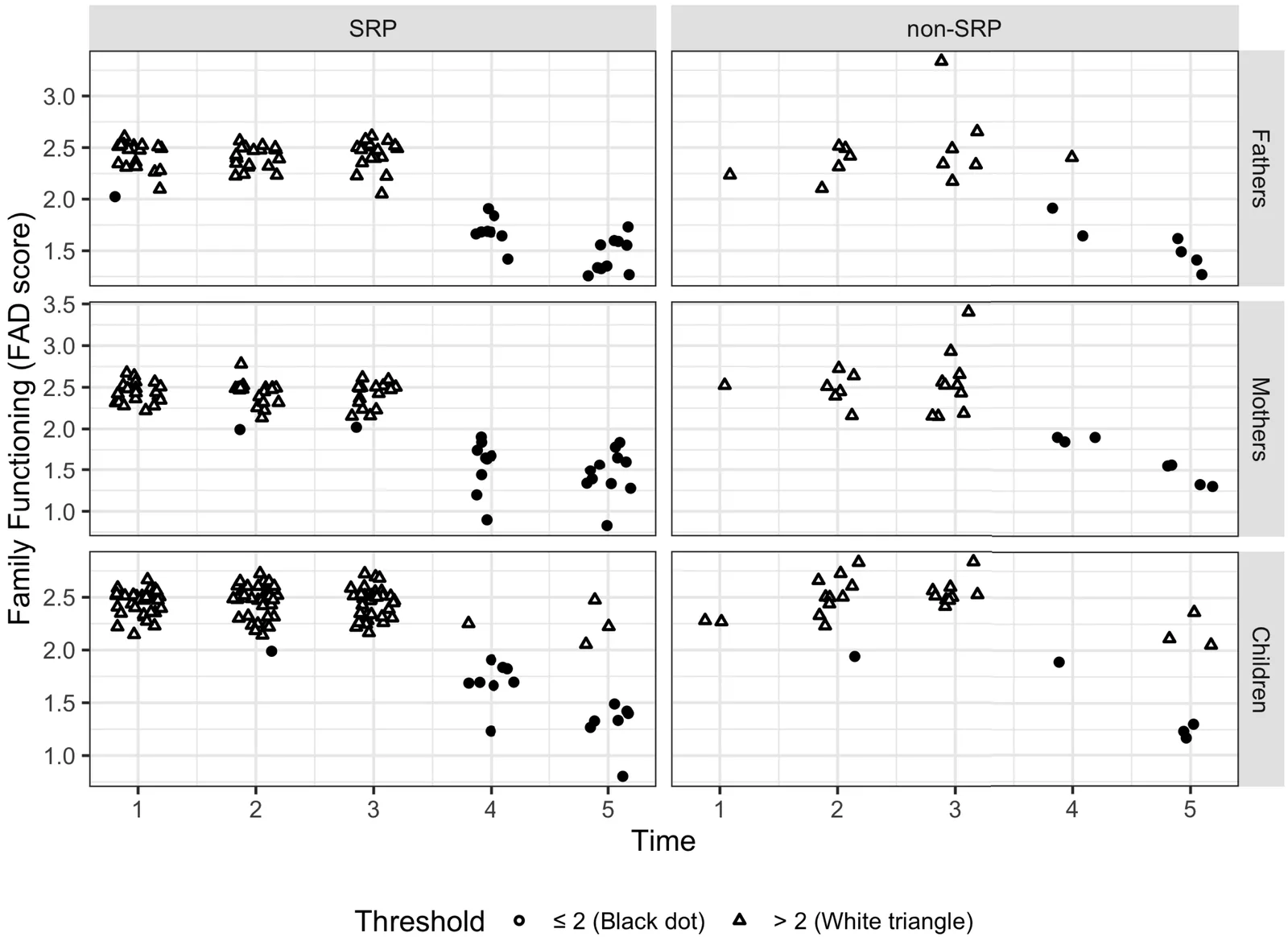
Individual Family Assessment Device (FAD) scores across time. Higher scores indicate more problematic family functioning; scores at or above 2 are shown relative to the reference value.

Fig. 3 displays individual FAD scores over time relative to the reference value of 2. The scatterplot is broadly consistent with the descriptive table, showing more scores below the reference value in later waves, but should not be used as the sole basis for claims about group differences or change over time.

Overall, the descriptive means, exploratory mean comparisons, and threshold comparisons suggest that perceived family functioning became less problematic over time in both SRP and non-SRP families. Nevertheless, given the small and uneven subgroup sizes, unequal follow-up, and limited precision of subgroup estimates, these patterns should not be interpreted as statistically confirmed improvements.

### 3.6 Exploratory longitudinal models

As a secondary exploratory analysis, we used hierarchical linear modeling (HLM) to examine patterns in SF-12 MCS scores while accounting for the nested structure of the data. The dependent variable in all hierarchical linear models was the SF-12 MCS score. These models therefore describe variation in MHQoL, not MINI-based psychiatric disorder prevalence. The variables used in the analyses, their scoring direction, and their interpretation are summarized in Appendix Table 7.

The models included random intercepts for family and for participants nested within family, thereby accounting for repeated observations within participants as well as clustering of participants within families. Given the small sample size, unequal group sizes, irregular follow-up intervals, and missing observations across waves, model estimates, convergence behavior (including singular fits in some specifications), and likelihood-ratio tests should be interpreted cautiously as descriptive rather than confirmatory.

Results from model comparisons are summarized in Table 6, with detailed parameter estimates reported in Appendix Table 12.

The null model included random intercepts for family and participant nested within family. Model 1 added migration pathway, family role, centered assessment wave (Time_c), quadratic time (${{\mathrm{Time}}_{c}}^{2}$), and physical health-related quality of life as predictors. Model 2 added interactions between migration pathway and the linear and quadratic time terms to explore whether temporal patterns differed between SRP and non-SRP participants. Model 3 added family functioning, measured using FAD scores, and Model 4 added interactions between FAD scores and the time terms.

**Table 6:** Fit of exploratory SF-12 MCS models of MHQoL.

Model	AIC	BIC	df	Marginal $R^{2}$	Conditional $R^{2}$
Null model	1933.30	1947.40	4	0.000	0.215
Model 1	1898.04	1933.30	10	0.187	0.304
Model 2	1890.51	1932.82	12	0.226	0.342
Model 3	1892.06	1937.89	13	0.228	0.342
Model 4	1894.83	1947.71	15	0.232	0.349

*Note.* The dependent variable was the SF-12 MCS score. Higher scores indicate better MHQoL. All models used 251 observations. df = number of model parameters, not residual degrees of freedom. Models included random intercepts for family and participant nested within family. Marginal $R^{2}$ describes variance explained by fixed effects; conditional $R^{2}$ describes variance explained by fixed and random effects. Model 4 produced a singular fit and is reported for transparency but should not be interpreted substantively.

Likelihood-ratio tests indicated that Model 1 fit the data better than the null model ($\Delta \chi^{2}\left(6\right)=47.26$, $p=1.66\times 10^{-8}$). Model 2 further improved fit relative to Model 1 ($\Delta \chi^{2}\left(2\right)=11.53$, p=0.003) and showed the lowest AIC and BIC values. This suggests that allowing temporal patterns to differ between SRP and non-SRP participants provided the best fit among the tested exploratory specifications based on AIC/BIC, while remaining subject to uncertainty in model selection.

Adding FAD scores in Model 3 did not improve model fit relative to Model 2 ($\Delta \chi^{2}\left(1\right)=0.45$, p=0.501). Adding FAD-by-time interactions in Model 4 also did not improve model fit relative to Model 3 ($\Delta \chi^{2}\left(2\right)=1.23$, p=0.540), and Model 4 produced a singular fit. Therefore, Model 4 was retained only for transparency and was not interpreted substantively.

As a sensitivity analysis, Models 2 and 4 were re-estimated treating assessment wave as a categorical factor rather than using centered linear and quadratic time terms. The categorical-time models produced similar estimates of explained variance and did not materially improve model fit despite the additional model parameters Appendix Table 15. These findings indicate that the substantive conclusions were robust to the specification of time.

**Fig. 4:**
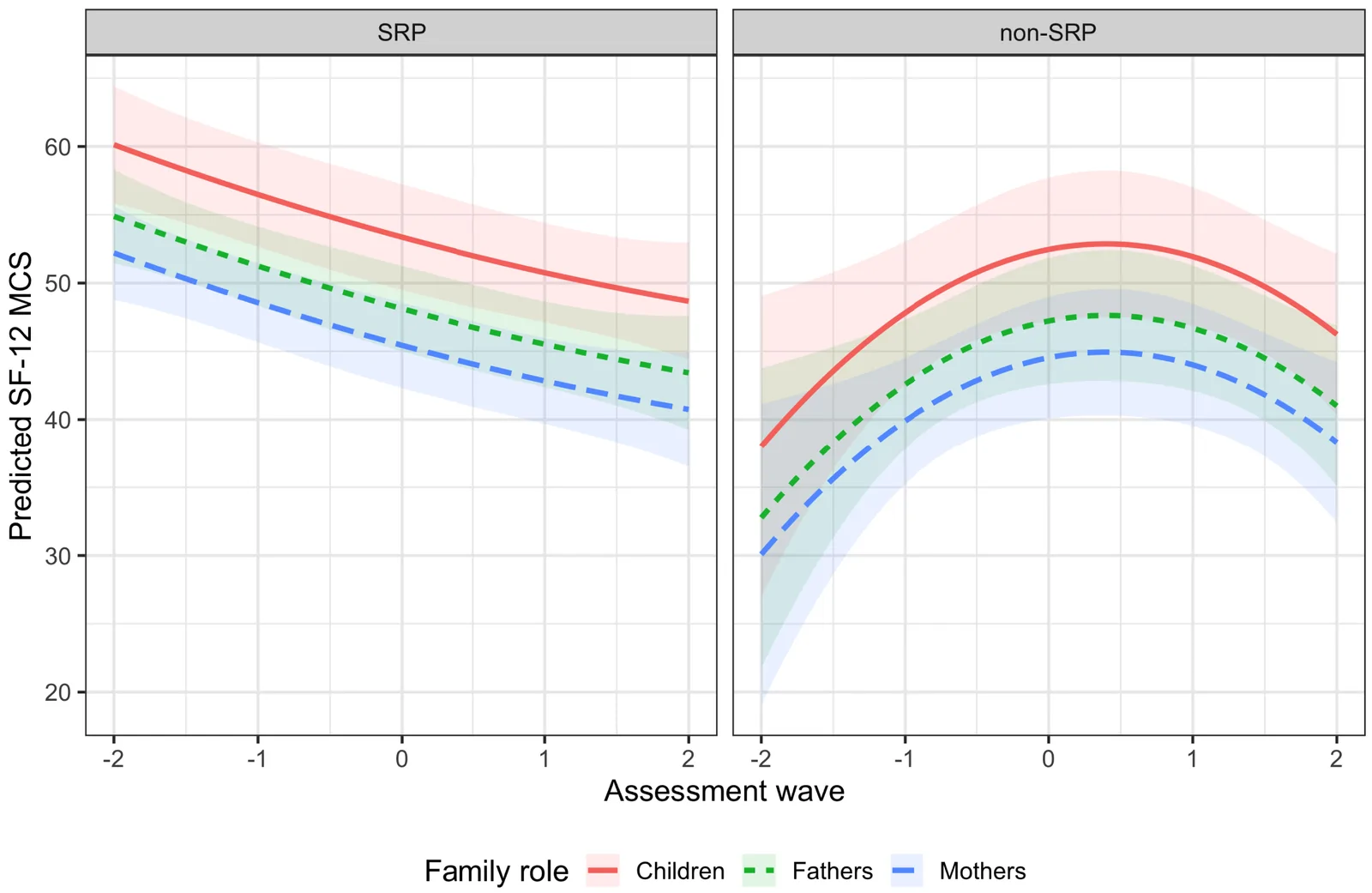
Predicted SF-12 mental component summary trajectories in SRP and non-SRP families based on exploratory Model 2. Higher scores indicate better MHQoL. Predictions are based on fixed effects from the model including migration pathway-by-time interactions, family role, and physical health-related functioning.

Fig. 4 displays the predicted SF-12 MCS trajectories from Model 2. These predicted trajectories should be interpreted as model-based summaries of the observed data rather than as definitive estimates of causal or population-level effects.

SRP and non-SRP participants showed different model-implied patterns in SF-12 MCS scores over time, although these patterns should be interpreted cautiously given sample size limitations and model instability in some specifications. Children generally had higher predicted scores than adults. Across the adjusted models, mothers reported significantly lower SF-12 MCS scores than children, while fathers also reported lower scores than children, although the magnitude of these differences was smaller. These findings should nevertheless be interpreted cautiously because of the exploratory design, modest sample size, and limited precision of some subgroup estimates

Overall, the exploratory models were broadly consistent with the descriptive analyses, while providing additional evidence that MHQoL trajectories differed by migration pathway over time. The improvement from Model 1 to Model 2 indicates that pathway-specific trajectories better represented the observed data than a single common time trend. Family functioning did not improve model fit or explain variation in MHQoL trajectories after adjustment. These findings should be interpreted as exploratory and should not be considered evidence of causal effects or definitive group differences.

A sensitivity analysis excluding the two earliest non-SRP observations (one father and one mother at Time 1) yielded substantively similar fixed-effect estimates and predicted trajectories Appendix Table 16. The associations between family role and MHQoL remained essentially unchanged, and the migration pathway-by-time interaction showed only modest attenuation, indicating that the primary findings were not driven by these sparse baseline observations.

Additional sensitivity analyses supported the primary findings. In the 36-month truncated sample (see Appendix Table 13), Model 2 again improved fit relative to Model 1 and retained the lowest AIC among the principal candidate models, although BIC slightly favored the simpler model. Similarly, in the married-couples sample (see Appendix Table 14), Model 2 improved fit relative to Model 1 and showed the lowest AIC, with BIC providing similar support for Models 1 and 2. Across both sensitivity analyses, migration pathway-by-time interactions remained evident, mothers consistently reported lower MHQoL than children, and family functioning did not materially improve model fit. As in the primary analyses, several extended models produced boundary (singular) fits, indicating limited parameter stability and supporting cautious interpretation.

## 4 Discussion

This exploratory longitudinal study described patterns of MHQoL, probable psychiatric disorder prevalence, and family functioning among Syrian forced migrant families living in the canton of Vaud, Switzerland. By including fathers, mothers, and children across repeated assessments, the study provides rare family-level longitudinal evidence on mental health among forcibly displaced families in a European host context. Given the exploratory design, the findings should be interpreted as hypothesis-generating rather than confirmatory.

A first descriptive finding was the substantial burden of probable psychiatric disorders among adults in both SRP and non-SRP families. High proportions of fathers and mothers met criteria for probable depression, PTSD, panic disorder, or anxiety-related disorders, consistent with previous research documenting elevated psychological distress among forcibly displaced populations exposed to war, displacement, and post-migration stressors (Al Ibraheem et al., 2017, Dangmann et al., 2021). Children showed lower observed prevalence than adults across most disorders. This may reflect lower symptom burden, greater resilience, differences in symptom expression, or possible under-detection through available assessment tools (Daud et al., 2005). The lower prevalence observed among children should therefore be interpreted cautiously rather than as evidence of absence of need.

Descriptive and model-based patterns suggested heterogeneity in SF-12 MCS scores over time. The primary longitudinal model indicated that allowing migration pathway-specific trajectories improved model fit compared with a common trajectory for all participants. However, a sensitivity analysis excluding the two sparse non-SRP observations at the earliest assessment attenuated the migration pathway × time interaction, indicating that evidence for pathway-specific trajectories should be interpreted cautiously. Accordingly, these findings should be regarded as exploratory and hypothesis-generating.

These pathway-specific patterns should not be interpreted as causal effects of the SRP. The SRP and non-SRP groups were not exchangeable comparison groups. SRP families were selected according to vulnerability criteria and received structured resettlement support, whereas non-SRP families entered through a different legal and administrative pathway and were likely exposed to different forms of uncertainty before and after arrival. Observed group differences may therefore reflect pre-existing vulnerability, migration pathway, timing of observation, legal status, support structures, family composition, or unmeasured post-migration stressors. Consequently, the study cannot determine whether SRP support improved, protected, or failed to protect MHQoL.

Although the primary longitudinal models suggested differing MHQoL trajectories between migration pathways, this finding was attenuated in sensitivity analyses excluding the sparse baseline non-SRP observations. Consequently, any apparent pathway-specific trajectory should be interpreted cautiously given the small sample size, sparse baseline observations, irregular follow-up, and exploratory nature of the analyses (Tinghög et al., 2017, Laban et al., 2005).

Family functioning, measured using the FAD, descriptively showed lower FAD scores at later assessments in both SRP and non-SRP families. This descriptive improvement may reflect family adaptation, increased familiarity with the host context, or stabilization of living conditions. However, FAD scores did not improve the fit of the exploratory SF-12 MCS models after accounting for migration pathway, family role, time, and physical health-related quality of life. This finding should not be taken as evidence that family functioning is unimportant. Rather, the small sample size, limited statistical power, unequal follow-up, and possible measurement limitations may have limited the ability to detect effects. Structural and migration-related factors, such as legal status, housing, financial insecurity, and access to services, may also have played a stronger role in explaining variation in MHQoL in this sample.

Mothers consistently reported lower MHQoL than children across the adjusted longitudinal models, while fathers also reported lower MHQoL than children, although the magnitude of these differences was smaller. Descriptive analyses likewise showed a high prevalence of probable psychiatric disorders among mothers. Together, these findings are consistent with literature highlighting the psychological burden experienced by women in forced migration contexts, including caregiving responsibilities, cumulative trauma exposure, social isolation, and barriers to care (Tinghög et al., 2017, Bunn et al., 2022). At the same time, MINI/MINI-KID and SF-12 MCS assess different aspects of mental health. The MINI/MINI-KID provides categorical diagnostic classifications based on symptom criteria, whereas the SF-12 MCS measures continuous mental health-related quality of life. Accordingly, differences between disorder prevalence and MHQoL should not be interpreted as contradictory but rather as reflecting complementary dimensions of mental health. Despite the consistency of the observed mother-child differences, these findings remain exploratory and require confirmation in larger longitudinal studies.

Several limitations should be emphasized. First, the sample was small and drawn from one Swiss canton, which limits generalizability and statistical power. The non-SRP group was especially small and had very limited participation at Time 1, restricting direct baseline comparisons. Second, families were not randomly assigned to SRP or non-SRP pathways. SRP selection was based on vulnerability criteria and linked to structured support, while non-SRP families followed a different legal and administrative pathway. Observed differences between groups therefore cannot be interpreted as program effects or as evidence of an SRP advantage or disadvantage.

Third, follow-up intervals were not homogeneous across families, and not all participants completed every assessment wave. Assessment waves were used as the time variable in the main HLM analyses, but actual time since arrival and time between assessments varied across participants. Consequently, analyses were based on available observations, which may have introduced bias if loss to follow-up was related to unmeasured participant characteristics or mental health status. This limits wave-to-wave comparability and may obscure, attenuate, or exaggerate apparent changes over time. Sensitivity analyses excluding observations collected more than 36 months after arrival partly addressed delayed follow-up observations, but they could not fully resolve non-homogeneous assessment timing.

Fourth, the child analyses were limited by the age criterion for participation. Because only children older than eight years were eligible, the study does not capture the experiences of younger children, who may express distress through developmental, behavioral, or somatic symptoms not captured by instruments designed for older children and adolescents. Fifth, the hierarchical models should be interpreted cautiously. HLM and likelihood-ratio tests rely on large-sample assumptions, and several sensitivity models produced singular fits, indicating limited information for estimating all random-effect components.

Sixth, the SF-12 was originally developed for adults and has not been specifically validated for younger Syrian forcibly displaced children. Although the instrument provided a common measure of mental health-related quality of life across family members and questionnaires were administered with linguistic and cultural support, findings relating to children should be interpreted cautiously. In addition, neither the SF-12 nor the FAD has been formally validated in this specific sample of Syrian forcibly displaced families living in Switzerland. Consequently, scores may not fully capture culturally specific expressions of MHQoL or family functioning.

Despite these limitations, the study has several strengths. It followed Syrian forced migrant families prospectively over several years, included multiple family members rather than focusing only on individuals, and considered both probable psychiatric disorder prevalence and MHQoL. The findings highlight the dynamic and heterogeneous nature of mental health following forced displacement, including differing MHQoL trajectories by migration pathway and consistently lower MHQoL among mothers compared with children.

Overall, these exploratory findings support the importance of sustained, culturally responsive, and family-centered psychosocial support for forcibly displaced families. Rather than assuming that early resettlement assistance is sufficient, services should include repeated needs assessments, accessible mental health care, attention to parental well-being, and support for family adaptation over time. Future research should examine these patterns in larger and more diverse samples, use culturally validated instruments where possible, and investigate the mechanisms linking migration pathway, post-migration stressors, family processes, and mental health trajectories.

## 5 Conclusions

This exploratory longitudinal study described MHQoL, probable psychiatric disorder prevalence, and family functioning among Syrian forced migrant families in Switzerland. The findings suggest a substantial burden of probable psychiatric disorders among adults, lower observed prevalence among children, and heterogeneous MHQoL trajectories that differed by migration pathway over time. Descriptive and exploratory longitudinal analyses indicated differing MHQoL trajectories across migration pathways, while mothers consistently reported lower MHQoL than children. These findings should be interpreted cautiously given the small sample size, unequal group sizes, irregular follow-up intervals, and non-random allocation to SRP and non-SRP pathways.

The study does not allow causal conclusions about the Swiss Resettlement Program or the standard asylum process. Observed differences may reflect selection processes, pre-migration experiences, timing of arrival, legal pathways, support conditions, and post-migration stressors rather than program effects.

Despite these limitations, the findings point to the need for sustained attention to the mental health and family well-being of forcibly displaced families after arrival. Psychosocial support may need to extend beyond the early resettlement period and include repeated needs assessments, accessible mental health care, attention to parental well-being, and support for family adaptation and social connection.

Future research should examine these patterns in larger and more diverse samples, use culturally validated instruments where possible, and include gender-, legal-, and migration-related variables to better understand how post-migration stressors, support structures, and family processes shape mental health and well-being among refugee and asylum-seeking families.

## CRediT authorship contribution statement

**Kevin Morisod:** Writing – review & editing, Writing – original draft, Methodology, Formal analysis, Data curation. **Khoa Nguyen:** Writing – review & editing, Writing – original draft, Visualization, Methodology, Formal analysis, Data curation. **Véronique S. Grazioli:** Writing – review & editing, Methodology, Conceptualization. **Dario Spini:** Writing – review & editing, Project administration, Methodology, Funding acquisition, Conceptualization. **Elie Abboche:** Writing – review & editing, Data curation. **Chahnaz Abdulghafour:** Writing – review & editing, Investigation. **Patrick Bodenmann:** Writing – review & editing, Project administration, Methodology, Funding acquisition, Conceptualization.

## Code availability

The R scripts used for statistical analyses, sensitivity analyses, and figure generation are publicly available in the project’s GitHub repository at: https://github.com/khoanguyen-dev/syrian-family-longitudinal-analysis.git

## Ethics declaration

Written informed consent to take part in the study and to publish the article has been obtained from all participants or their legal representatives. The privacy rights of participants have been observed.

This study was performed in compliance with relevant laws, regulatory frameworks and guidelines where the research took place. This study was approved by the Swiss Ethics Committees. (Approval No. 2018–00925)

All procedures were approved by the appropriate ethics committee. Participants were informed of their right to withdraw from the study at any time without affecting their access to healthcare or support services. Data were anonymized and securely stored, with access restricted to authorized research personnel.

## Declaration of generative AI and AI-assisted technologies in the manuscript preparation process

During the preparation of this work the authors used Perplexity, ChatGPT in order to refine the wording and improve the structure of the manuscript. After using these tools, the authors reviewed and edited the content as needed and take full responsibility for the content of the published article.

## Funding

This study was mainly funded by the public health service of the canton of Vaud, through a fund dedicated to prevention, promotion of health, and against addictions (CPSLA, canton of Vaud, funding number 8273/3634000000-811). It also received a financial contribution from the Center LIVES , the Swiss Center of Expertise in Life Course Research, University of Lausanne and Geneva.

## Declaration of competing interest

The authors declare that they have no known competing financial interests or personal relationships that could have appeared to influence the work reported in this paper.

## Figures and Tables

**Table 7:** Outcome variables, scoring direction, and role in analyses.

Measure	Variable used	Scoring direction	Interpretation and role in analysis
MINI/MINI-Kid	Probable psychiatric disorder diagnoses (major depressive disorder, panic disorder, PTSD, and generalized or separation anxiety)	Binary indicator: probable diagnosis present vs. absent	Used to describe prevalence by migration pathway and family role. Indicators reflect whether structured diagnostic criteria were met according to MINI/MINI-KID algorithms. These variables were not used as dependent variables in longitudinal HLM models.

SF-12 Mental Component Summary (MCS)	Mental health-related quality of life	Higher scores indicate better mental health-related quality of life	Main dependent variable in the exploratory longitudinal HLM models. Scores at or below the reference value of 50 were interpreted as indicating poorer-than-average mental health-related quality of life relative to the reference value.

SF-12 Physical Component Summary (PCS)	Physical component of health-related quality of life	Higher scores indicate better physical health-related quality of life	Included as a covariate in the exploratory longitudinal models to account for physical health-related quality of life.

Family Assessment Device (FAD)	Perceived family functioning	Higher scores indicate more problematic family functioning	Used to describe perceived family functioning and as an exploratory predictor in the longitudinal models. Scores at or above 2 were interpreted as indicating more problematic family functioning relative to the reference value.

*Note:* MINI = Mini International Neuropsychiatric Interview; SF-12 = 12-item Short Form Health Survey; MCS = Mental Component Summary; PCS = Physical Component Summary; FAD = Family Assessment Device; PTSD = post-traumatic stress disorder; HLM = hierarchical linear modeling.

**Table 8:** Exploratory mean comparisons of SF-12 MCS scores between SRP and non-SRP participants.

Time	Role	SRP n	Mean SRP	Non-SRP n	Mean non-SRP	Mean diff.	p
T1	Child	9	57.49	0	–	–	–
T1	Father	19	56.37	1	22.12	34.25	–
T1	Mother	19	53.17	1	31.03	22.14	–
T2	Child	9	51.89	4	45.41	6.48	0.336
T2	Father	19	52.24	5	40.11	12.13	0.099
T2	Mother	19	47.29	7	47.23	0.06	0.991
T3	Child	11	54.27	3	56.66	−2.39	0.340
T3	Father	17	51.24	5	45.44	5.79	0.569
T3	Mother	17	42.43	9	42.59	−0.16	0.979
T4	Child	11	57.92	1	42.72	15.20	–
T4	Father	8	43.70	3	54.14	−10.44	0.410
T4	Mother	10	40.95	3	45.35	−4.40	0.499
T5	Child	13	51.53	6	52.28	−0.76	0.864
T5	Father	11	39.03	4	33.89	5.14	0.419
T5	Mother	12	41.76	5	41.06	0.69	0.786

*Note:* Mean difference = SRP mean minus non-SRP mean. Positive values indicate higher SF-12 MCS scores among SRP participants. Exploratory Welch’s t-tests were performed only when both migration pathway groups contained at least three observations. Cells with fewer observations are reported descriptively without inferential testing.

**Table 9:** Exploratory threshold comparisons for SF-12 MCS scores at or below 50.

Time	Role	SRP n	SRP ≤ 50	Non-SRP n	Non-SRP ≤ 50	Diff. pp	Fisher p
T1	Child	9	1(11.1%)	0	–	–	–
T1	Father	19	3(15.8%)	1	1(100.0%)	−84.2	–
T1	Mother	19	5(26.3%)	1	1(100.0%)	−73.7	–
T2	Child	9	4(44.4%)	4	3(75.0%)	−30.6	0.559
T2	Father	19	7(36.8%)	5	4(80.0%)	−43.2	0.142
T2	Mother	19	9(47.4%)	7	3(42.9%)	4.5	1.000
T3	Child	11	3(27.3%)	3	0(0.0%)	27.3	1.000
T3	Father	17	6(35.3%)	5	2(40.0%)	−4.7	1.000
T3	Mother	17	11(64.7%)	9	5(55.6%)	9.2	0.692
T4	Child	11	1(9.1%)	1	1(100.0%)	−90.9	–
T4	Father	8	6(75.0%)	3	1(33.3%)	41.7	0.491
T4	Mother	10	9(90.0%)	3	2(66.7%)	23.3	0.423
T5	Child	13	6(46.2%)	6	3(50.0%)	−3.8	1.000
T5	Father	11	10(90.9%)	4	4(100.0%)	−9.1	1.000
T5	Mother	12	11(91.7%)	5	5(100.0%)	−8.3	1.000

*Note:* Values show the number and percentage of participants with SF-12 MCS scores at or below 50. Diff. pp = difference in percentage points, calculated as SRP minus non-SRP. Exploratory Fisher’s exact tests were performed only when both migration pathway groups contained at least three observations. Cells with fewer observations are reported descriptively without inferential testing. Negative values indicate a higher proportion at or below 50 in the non-SRP group. Fisher’s exact tests were used because of small cell counts. The value of 50 was used as a reference value, not as a clinical diagnostic threshold.

**Table 10:** Exploratory mean comparisons of FAD scores between SRP and non-SRP participants.

Time	Role	SRP n	Mean SRP	Non-SRP n	Mean non-SRP	Mean diff.	p
T1	Child	33	2.45	2	2.25	0.20	–
T1	Father	19	2.39	1	2.25	0.15	–
T1	Mother	19	2.43	1	2.50	−0.07	–
T2	Child	38	2.46	11	2.48	−0.02	0.798
T2	Father	19	2.42	5	2.37	0.05	0.565
T2	Mother	19	2.42	6	2.49	−0.07	0.477
T3	Child	33	2.45	9	2.55	−0.10	0.050
T3	Father	17	2.44	6	2.56	−0.12	0.520
T3	Mother	17	2.39	10	2.55	−0.16	0.236
T4	Child	9	1.75	1	1.92	−0.17	–
T4	Father	8	1.69	3	2.00	−0.31	0.290
T4	Mother	9	1.56	3	1.89	−0.33	0.016
T5	Child	10	1.59	6	1.71	−0.12	0.669
T5	Father	10	1.46	4	1.44	0.02	0.825
T5	Mother	11	1.46	4	1.46	0.00	0.973

*Note:* Mean difference = SRP mean minus non-SRP mean. Negative values indicate lower, or less problematic, FAD scores among SRP participants. Welch’s tests were used for exploratory comparisons only when both migration pathway groups contained at least three observations. Comparisons not meeting this criterion are presented descriptively without inferential statistics. Estimates should be interpreted cautiously because subgroup sizes were small and unequal, particularly in the non-SRP group.

**Table 11:** Exploratory threshold comparisons for FAD scores at or above 2

Time	Role	SRP n	SRP ≥ 2	Non-SRP n	Non-SRP ≥ 2	Diff. pp	Fisher p
T1	Child	33	33(100.0%)	2	2(100.0%)	0.0	–
T1	Father	19	19(100.0%)	1	1(100.0%)	0.0	–
T1	Mother	19	19(100.0%)	1	1(100.0%)	0.0	–
T2	Child	38	38(100.0%)	11	10(90.9%)	9.1	0.224
T2	Father	19	19(100.0%)	5	5(100.0%)	0.0	1.000
T2	Mother	19	19(100.0%)	6	6(100.0%)	0.0	1.000
T3	Child	33	33(100.0%)	9	9(100.0%)	0.0	1.000
T3	Father	17	17(100.0%)	6	6(100.0%)	0.0	1.000
T3	Mother	17	17(100.0%)	10	10(100.0%)	0.0	1.000
T4	Child	9	1(11.1%)	1	0(0.0%)	11.1	–
T4	Father	8	0(0.0%)	3	1(33.3%)	−33.3	0.273
T4	Mother	9	0(0.0%)	3	0(0.0%)	0.0	1.000
T5	Child	10	3(30.0%)	6	3(50.0%)	−20.0	0.607
T5	Father	10	0(0.0%)	4	0(0.0%)	0.0	1.000
T5	Mother	11	0(0.0%)	4	0(0.0%)	0.0	1.000

*Note:* Values show the number and percentage of participants with FAD scores at or above 2. Diff. pp = difference in percentage points, calculated as SRP minus non-SRP. Positive values indicate a higher proportion at or above 2 among SRP participants. Fisher’s exact tests were used because of small cell counts. The value of 2 was used as a reference value indicating more problematic family functioning.

**Table 12:** Summary of HLM Results for SF-12 MCS.

Parameter	Null			Model 1			Model 2			Model 3			Model 4		
	Est.	95% CI	p	Est.	95% CI	p	Est.	95% CI	p	Est.	95% CI	p	Est.	95% CI	p
Intercept	47.86	(45.63, 50.10)	<0.001	48.60	(40.87, 56.33)	<0.001	50.06	(42.43, 57.68)	<0.001	53.87	(40.32, 67.41)	<0.001	57.11	(41.40, 72.83)	<0.001
Non-SRP group				−4.09	(−8.41, 0.24)	0.063	−0.90	(−6.10, 4.29)	0.730	−0.75	(−5.96, 4.46)	0.775	−0.79	(−6.05, 4.46)	0.765
Fathers vs. children				−4.79	(−8.55, −1.04)	0.013	−5.24	(−8.95, −1.53)	0.006	−5.36	(−9.08, −1.65)	0.005	−5.50	(−9.15, −1.85)	0.003
Mothers vs. children				−7.39	(−11.18, −3.60)	<0.001	−7.93	(−11.68, −4.17)	<0.001	−8.04	(−11.79, −4.28)	<0.001	−8.09	(−11.77, −4.41)	<0.001
Physical health				0.11	(−0.02, 0.24)	0.103	0.08	(−0.05, 0.20)	0.243	0.08	(−0.05, 0.21)	0.216	0.08	(−0.05, 0.20)	0.241
Time				−2.32	(−3.28, −1.37)	<0.001	−2.86	(−3.90, −1.83)	<0.001	−3.30	(−4.95, −1.65)	<0.001	−8.48	(−18.83, 1.88)	0.108
Time${}^{2}$				0.19	(−0.55, 0.94)	0.612	0.26	(−0.54, 1.06)	0.525	0.10	(−0.82, 1.03)	0.823	0.86	(−4.67, 6.39)	0.760
Non-SRP group: Time							4.92	(2.01, 7.83)	0.001	5.02	(2.10, 7.94)	0.001	4.98	(2.06, 7.91)	0.001
Non-SRP group: Time${}^{2}$							−2.84	(−5.13, −0.56)	0.015	−2.87	(−5.15, −0.59)	0.014	−2.88	(−5.16, −0.59)	0.014
FAD										−1.71	(−6.72, 3.30)	0.501	−2.91	(−8.92, 3.11)	0.342
FAD: Time													2.37	(−2.13, 6.88)	0.300
FAD: Time${}^{2}$													−0.19	(−2.59, 2.21)	0.875

Variance components															

Residual variance	105.32			94.04			89.13			89.16			88.80		
Family random intercept variance	16.51			15.70			14.62			14.73			15.97		
Participant-within-family random intercept variance	12.35			0.05			1.07			0.77			0.00		
Family ICC	0.123			0.143			0.139			0.141			0.152		
Participant-within-family ICC	0.092			0.000			0.010			0.007			0.000		
Total random intercept ICC	0.215			0.143			0.150			0.148			0.152		

Pseudo $R^{2}$															

Marginal $R^{2}$	0.000			0.187			0.226			0.228			0.232		
Conditional $R^{2}$	0.215			0.304			0.342			0.342			0.349		

*Note.* The dependent variable was the SF-12 MCS score, with higher scores indicating better mental health-related quality of life. SRP status and family role were coded using SRP families and children as the reference categories, respectively. All models included random intercepts for family and participant nested within family. Several models produced boundary (singular) fits, with participant-level variance estimated as zero in the more complex models, indicating limited information for estimating participant-specific random effects within this restricted sample. Marginal $R^{2}$ represents the variance explained by fixed effects, whereas conditional $R^{2}$ represents the variance explained by both fixed and random effects.

**Table 13:** Summary of HLM Results for SF-12 MCS with only the Information Collected during the First 36 Months after Participants’ Arrival in the Canton was Used (SE = Standard Error)

Parameter	Null			Model 1			Model 2			Model 3			Model 4		
	Est.	95% CI	p	Est.	95% CI	p	Est.	95% CI	p	Est.	95% CI	p	Est.	95% CI	p
Intercept	49.61	(46.90, 52.32)	<0.001	46.85	(37.87, 55.83)	<0.001	46.39	(37.72, 55.06)	<0.001	49.97	(30.26, 69.67)	<0.001	68.15	(38.71, 97.59)	<0.001
Non-SRP group				−3.97	(−9.86, 1.92)	0.181	0.01	(−7.61, 7.62)	0.999	0.44	(−7.46, 8.33)	0.913	0.33	(−7.79, 8.45)	0.936
Fathers vs. children				−2.07	(−6.27, 2.14)	0.334	−2.00	(−6.12, 2.12)	0.339	−2.09	(−6.23, 2.05)	0.321	−2.42	(−6.53, 1.69)	0.247
Mothers vs. children				−6.49	(−10.81, −2.17)	0.003	−6.52	(−10.76, −2.29)	0.003	−6.61	(−10.87, −2.36)	0.002	−6.96	(−11.17, −2.75)	0.001
Physical health				0.11	(−0.03, 0.26)	0.127	0.11	(−0.03, 0.26)	0.118	0.12	(−0.03, 0.26)	0.115	0.10	(−0.04, 0.24)	0.159
Time				−1.48	(−3.91, 0.95)	0.232	−1.43	(−3.86, 1.00)	0.247	−2.04	(−5.92, 1.84)	0.301	−23.34	(−41.39, −5.30)	0.012
Time${}^{2}$				0.76	(−0.74, 2.26)	0.317	1.04	(−0.47, 2.55)	0.178	0.76	(−1.28, 2.80)	0.464	−13.31	(−31.05, 4.43)	0.141
Non-SRP group: Time							−4.40	(−20.75, 11.95)	0.596	−3.47	(−20.44, 13.50)	0.687	−5.70	(−22.94, 11.54)	0.515
Non-SRP group: Time${}^{2}$							−8.08	(−17.44, 1.29)	0.090	−7.72	(−17.25, 1.81)	0.112	−8.78	(−18.36, 0.79)	0.072
FAD										−1.53	(−9.11, 6.05)	0.691	−8.22	(−19.81, 3.37)	0.163
FAD: Time													10.28	(1.75, 18.80)	0.018
FAD: Time${}^{2}$													6.47	(−1.07, 14.01)	0.092

Variance components															

Residual variance	90.37			81.18			80.70			80.63			76.93		
Family random intercept variance	25.72			25.42			17.00			17.00			19.98		
Participant-within-family random intercept variance	11.21			0.00			0.00			0.00			0.00		
Family ICC	0.202			0.238			0.174			0.174			0.206		
Participant-within-family ICC	0.088			0.000			0.000			0.000			0.000		
Total random intercept ICC	0.290			0.238			0.174			0.174			0.206		

Pseudo $R^{2}$															

Marginal $R^{2}$	0.000			0.155			0.199			0.200			0.217		
Conditional $R^{2}$	0.290			0.356			0.338			0.339			0.378		

*Note.* The dependent variable was the SF-12 MCS score, with higher scores indicating better mental health-related quality of life. This sensitivity analysis was restricted to observations collected within 36 months of arrival in the Canton. SRP status and family role were coded using SRP families and children as the reference categories, respectively. All models included random intercepts for family and participant nested within family. Several models produced boundary (singular) fits, with participant-level variance estimated as zero in the more complex models, indicating limited information for estimating participant-specific random effects within this restricted sample. Marginal $R^{2}$ represents the variance explained by fixed effects, whereas conditional $R^{2}$ represents the variance explained by both fixed and random effects.

**Table 14:** Summary of HLM Results for SF-12 MCS Restricted to Married-Couple Families.

Parameter	Null			Model 1			Model 2			Model 3			Model 4		
	Est.	95% CI	p	Est.	95% CI	p	Est.	95% CI	p	Est.	95% CI	p	Est.	95% CI	p
Intercept	48.21	(45.65, 50.76)	<0.001	49.93	(41.14, 58.72)	<0.001	51.04	(42.33, 59.75)	<0.001	49.64	(34.15, 65.14)	<0.001	53.38	(36.63, 70.13)	<0.001
Non-SRP group				−3.68	(−8.97, 1.61)	0.165	0.12	(−6.10, 6.34)	0.969	0.09	(−6.13, 6.31)	0.977	−0.04	(−6.39, 6.31)	0.990
Fathers vs. children				−6.83	(−10.99, −2.66)	0.001	−7.14	(−11.20, −3.07)	0.001	−7.09	(−11.18, −3.01)	0.001	−7.11	(−11.22, −3.00)	0.001
Mothers vs. children				−9.73	(−14.06, −5.39)	<0.001	−10.07	(−14.30, −5.84)	<0.001	−10.03	(−14.28, −5.79)	<0.001	−9.93	(−14.18, −5.69)	<0.001
Physical health				0.12	(−0.02, 0.27)	0.092	0.09	(−0.05, 0.24)	0.190	0.09	(−0.05, 0.23)	0.198	0.09	(−0.05, 0.23)	0.195
Time				−2.11	(−3.12, −1.10)	<0.001	−2.52	(−3.58, −1.45)	<0.001	−2.35	(−4.19, −0.52)	0.012	−8.09	(−19.66, 3.49)	0.170
Time^2^				0.16	(−0.64, 0.95)	0.698	0.32	(−0.51, 1.14)	0.452	0.37	(−0.61, 1.35)	0.453	0.58	(−5.18, 6.34)	0.842
Non-SRP group: Time							4.76	(1.55, 7.97)	0.004	4.73	(1.50, 7.95)	0.004	4.66	(1.44, 7.88)	0.005
Non-SRP group: Time^2^							−3.39	(−6.03, −0.74)	0.012	−3.39	(−6.03, −0.75)	0.012	−3.52	(−6.16, −0.87)	0.010
FAD										0.62	(−5.07, 6.31)	0.830	−0.89	(−7.26, 5.48)	0.783
FAD: Time													2.76	(−2.35, 7.86)	0.289
FAD: Time^2^													0.12	(−2.43, 2.67)	0.927

Variance components															

Residual variance	100.97			88.87			83.87			83.86			82.50		
Family random intercept variance	16.64			17.50			18.46			18.44			20.63		
Participant-within-family random intercept variance	15.38			0.00			0.00			0.00			0.00		
Family ICC	0.125			0.165			0.180			0.180			0.200		
Participant-within-family ICC	0.116			0.000			0.000			0.000			0.000		
Total random intercept ICC	0.241			0.165			0.180			0.180			0.200		

Pseudo $R^{2}$															

Marginal $R^{2}$	0.000			0.205			0.247			0.247			0.251		
Conditional $R^{2}$	0.241			0.336			0.383			0.383			0.401		

*Note.* The dependent variable was the SF-12 MCS score, with higher scores indicating better mental health-related quality of life. This sensitivity analysis was restricted to families in which both parents were married at baseline. SRP status and family role were coded using SRP families and children as the reference categories, respectively. All models included random intercepts for family and participant nested within family. Several models produced boundary (singular) fits, with the participant-level random-intercept variance estimated as zero in Models 1–4, reflecting limited information for estimating participant-level random effects in this restricted sample. Marginal $R^{2}$ represents the variance explained by the fixed effects, whereas conditional $R^{2}$ represents the variance explained by both the fixed and random effects.

**Table 15:** Sensitivity analysis using categorical assessment wave.

Fit statistic	Model 2	Model 2	Model 4	Model 4
	quadratic	categorical	quadratic	categorical
AIC	1890.51	1895.73	1894.83	1897.94
BIC	1932.82	1952.14	1947.71	1971.97
Number of parameters	12	16	15	21
Marginal $R^{2}$	0.226	0.234	0.232	0.255
Conditional $R^{2}$	0.342	0.349	0.349	0.369
Singular fit	No	No	Yes	No

*Note.* As a sensitivity analysis, assessment wave was alternatively modeled as a five-level categorical factor rather than using centered linear and quadratic time terms. Model 2 included migration pathway, family role, physical health-related quality of life, and migration pathway-by-time interactions. Model 4 additionally included family functioning (FAD) and FAD-by-time interactions. Although the categorical-time models included additional parameters and therefore showed slightly higher AIC and BIC values, the overall pattern of model fit and variance explained was similar to that of the primary quadratic-time models, indicating that the substantive conclusions were robust to the specification of time.

**Table 16:** Sensitivity analysis excluding the two earliest non-SRP observations.

Parameter	Original Model 2	Excluding non-SRP T1
Intercept	50.06 (<0.001)	49.94 (<0.001)
Non-SRP group	−0.90 (0.730)	−1.71 (0.530)
Fathers vs. children	−5.24 (0.006)	−5.06 (0.009)
Mothers vs. children	−7.93 (<0.001)	−7.83 (<0.001)
Physical health	0.08 (0.243)	0.08 (0.241)
Time	−2.86 (<0.001)	−2.86 (<0.001)
Time^2^	0.26 (0.525)	0.26 (0.523)
Non-SRP group × Time	4.92 (0.001)	3.43 (0.086)
Non-SRP group × Time^2^	−2.84 (0.015)	−1.80 (0.228)

AIC	1889.5	1875.7
Marginal $R^{2}$	0.226	0.206
Conditional $R^{2}$	0.342	0.327

*Note.* Two observations (one father and one mother from the non-SRP group at T1) were excluded because they represented sparse boundary observations. The sensitivity analysis produced substantively similar estimates for the family-role effects and overall trajectory pattern, indicating that the primary findings were not driven by these sparse baseline observations.
